# Notch-1 promotes the malignant progression of osteosarcoma through the activation of cell division cycle 20

**DOI:** 10.18632/aging.202314

**Published:** 2020-12-19

**Authors:** Yuan Gao, Lunhao Bai, Guanning Shang

**Affiliations:** 1 Department of Orthopedic Surgery, ShengJing Hospital, China Medical University, Liaoning, PR China; 2 Graduate School, China Medical University, Shenyang, Liaoning Province, PR China

**Keywords:** osteosarcoma, Cdc20, cell growth, migration, apoptosis

## Abstract

The molecular mechanism of osteosarcoma (OS) pathogenesis is poorly understood. The Notch signaling pathway has been shown to be critically involved in tumorigenesis, including OS. Therefore, we explored the molecular mechanism by which the Notch-1 signaling pathway is involved in OS progression. Several approaches were carried out to determine the biological function of Notch-1 in OS cells. The MTT results revealed that Notch-1 overexpression increased the viability of OS cells, whereas Notch-1 downregulation reduced cell viability. Consistently, modulation of Notch-1 regulated apoptosis and the migratory and invasive abilities of OS cells. Mechanistic studies showed that Notch-1 overexpression augmented cell division cycle 20 (Cdc20) expression in OS cells. Moreover, overexpression of Cdc20 alleviated the inhibitory effects of Notch-1 downregulation on the viability, migration and invasion of OS cells. Our study offers a promising OS treatment strategy by inhibiting Notch-1.

## INTRODUCTION

Osteosarcoma (OS) is a frequent aggressive bone tumor that occurs in children and young people [1, 2]. OS patients often experience metastasis to the bone marrow, brain and liver, leading to a worse prognosis [3]. In addition, during chemotherapeutic treatments, OS patients develop drug resistance [4]. The molecular mechanism of OS development and progression has not been fully elucidated [5]. Thus, exploration of OS pathogenesis is pivotal for the development of new treatment strategies to effectively fight OS. The Notch signaling pathway participates in development, differentiation and tumorigenesis [6–8]. It is known that mammals have four Notch receptors (Notch-1, Notch-2, Notch-3 and Notch-4) and five ligands (DLL-1, DLL-3, DLL-4, Jagged-1 and Jagged-2) [9]. Notch signaling is activated when the Notch receptor binds with its ligand, and subsequently, three proteolytic cleavages occur via several enzymes, including γ-secretase complex, and eventually produce intracellular domain of Notch (NICD), which activates its downstream targets [10, 11]. Therefore, gamma secretase inhibitors (GSIs) could inactivate Notch activation [12, 13].

The expression of Notch-1, Jagged-1, Hes-1 and Hey-2 was upregulated in OS tumor tissues [14]. Hes-4, a target of the Notch pathway, has been shown to be associated with poor survival in patients with high-grade OS [15]. The expression of Notch receptors has been investigated in human SaOS-2 OS cells. Increased expression of Notch-1 and weak expression of Notch-2 were observed in SaOS-2 cells [16]. Notch-3 was undetectable in SaOS-2 cells, while Notch-4 expression was detected after treatments with dexamethasone and vitamin D(3) in SaOS-2 cells [16]. This study indicated that Notch receptors were differentially expressed in OS cells, and Notch-1 might play a pivotal role in OS development [16]. However, Notch-2, Jagged-1, Hey-1, and Hey-2 were overexpressed in OS tumor biopsy specimens, while Notch-1 and DLL1 were decreased in these specimens [17]. High expression of Notch-3 in OS patient tumors is associated with metastasis and poor prognosis [18].

GSI treatment reduced the proliferation of OS cells *in vitro*, and chemical and genetic suppression of Notch slowed tumor growth in nude mice [14]. Similarly, GSI treatment slowed cell proliferation and tumor growth both *in vitro* and *in vivo* in OS [17]. GSI treatment also induced cell cycle arrest at G1 phase in OS cells via a reduction in the expression of several cell cycle accelerators, such as cyclin D1, E1, and E2 and Skp2 [17]. Conditional expression of NICD in immature osteoblasts in one mouse model led to bone tumors, including OS [19]. Moreover, the loss of p53 in combination with Notch activation accelerated OS occurrence in mice, indicating that Notch activation was a key driver of OS [19]. These reports reveal that the Notch pathway is critically involved in OS development.

Although Notch-1 has been shown to exert its oncogenic effects in OS, the detailed underlying mechanism has not been fully understood. Our previous studies have shown that cell division cycle 20 (Cdc20) promotes cell proliferation and motility in OS cells [20]. Inhibition of Cdc20 by its inhibitor Apcin suppressed viability, migration and invasion in OS cells [21]. In the present study, we explored the effects of Notch-1 on viability, apoptosis, migration and invasion in OS cells. Moreover, we determined whether Notch-1 exerts its biological effects via the upregulation of Cdc20 in OS cells. Our study might provide the rationale for a new therapeutic strategy by targeting the Notch-1 pathway in OS.

## RESULTS

### Inhibition of Notch-1 attenuates cell viability

**Figure 1:**
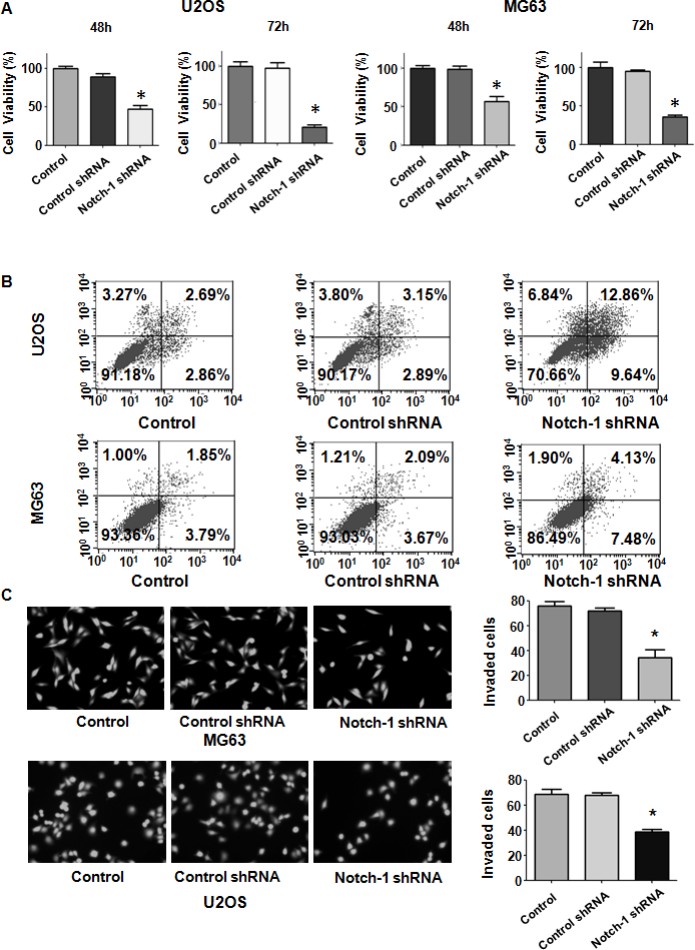
**Notch-1 shRNA transfection diminishes viability and stimulates apoptosis.** (**A**) Viability was evaluated by MTT assay. The MTT results demonstrated that the reduction in Notch-1 alleviated the viability of OS cells. *P < 0.05 vs control shRNA. (**B**) Apoptosis was examined by flow cytometry. Inhibition of Notch-1 led to increased apoptosis. (**C**) Transwell assays showed that Notch-1 shRNA treatment resulted in invasion retardation.

Evidence has shown that Notch-1 might participate in OS development and progression. To examine whether the modulation of Notch-1 expression levels affects the viability of OS cells, we performed MTT assays in U2OS and MG63 cells after Notch-1 shRNA transfection. Our MTT data showed that Notch-1 shRNA infection repressed viability in both U2OS and MG63 cells at 48 hours and 72 hours (Figure 1A). This result revealed that the inhibition of Notch-1 by shRNA transfection impaired the viability of OS cells.

### Inhibition of Notch-1 stimulates apoptosis

Since Notch-1 participates in the regulation of cell apoptosis, we examined whether the inhibition of Notch-1 affected apoptotic death in cells. OS cells were transfected with Notch-1 shRNA for 72 hours and then the PI-FITC-annexin assay was carried out to measure apoptosis rate in Notch-1 shRNA-transfected OS cells. We observed that the suppression of Notch-1 stimulated the apoptotic rate from 6% to 22.5% in U2OS cells after Notch-1 shRNA infection (Figure 1B). Similarly, Notch-1 shRNA transfection facilitated apoptosis from 5.7% to 11.6% in MG63 cells in the Notch-1 downregulation group (Figure 1B). Therefore, the inhibition of Notch-1 elevated OS cell apoptosis.

### Inhibition of Notch-1 represses migrative and invasive ability

**Figure 2:**
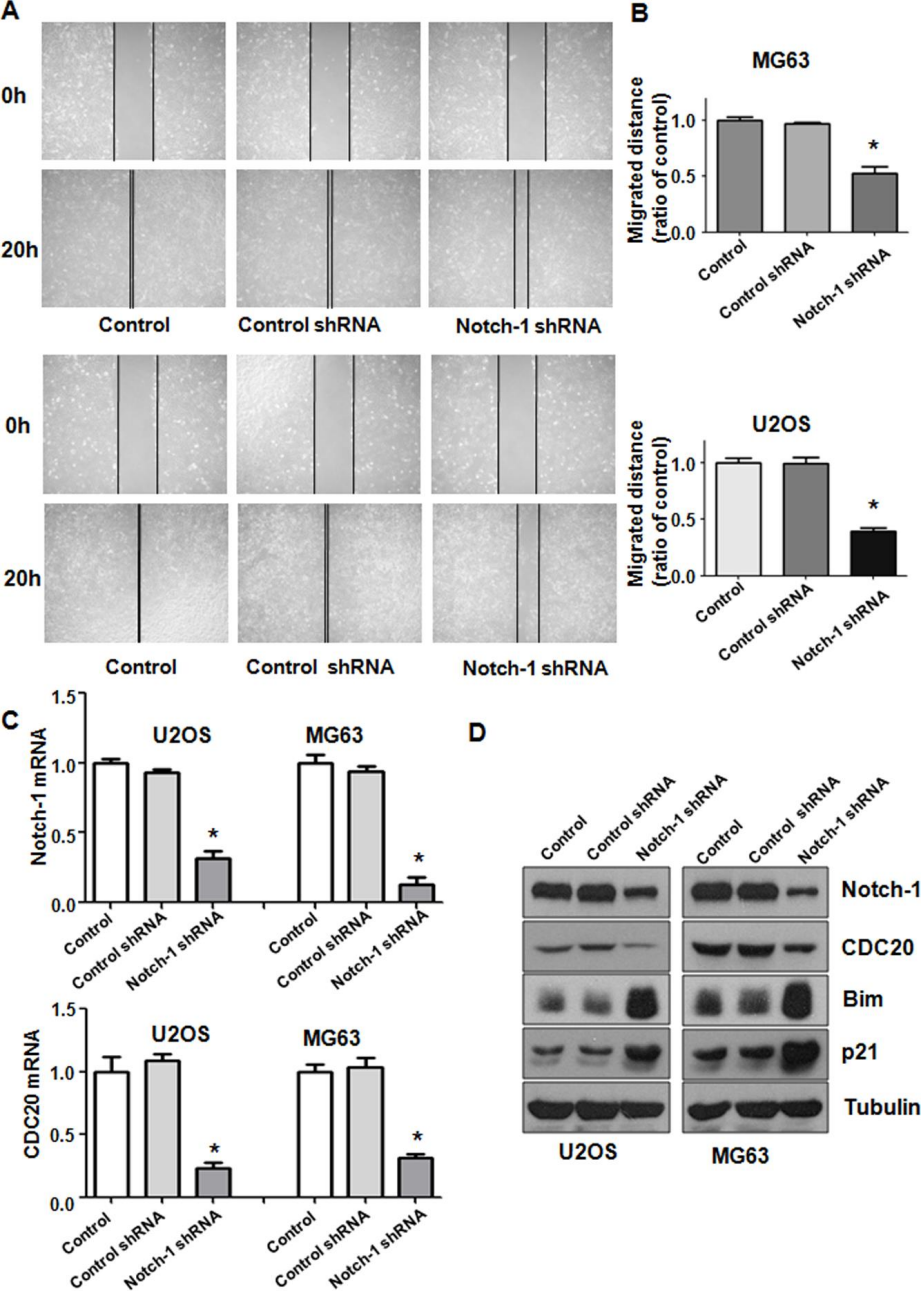
**Notch-1 shRNA alleviated Cdc20 expression.** (**A**) Wound healing assays showed that Notch-1 shRNA moderated wound closure. (**B**) Quantitative analysis f the migration data. *P < 0.05 vs control shRNA. (**C**) Real-time RT-PCR results showing Notch-1, Cdc20, Bim and p21 expression. (**D**) Western blot results showing Notch-1, Cdc20, Bim and p21 expression.

Notch-1 has been shown to regulate motility in cancer cells. Thus, we examined whether Notch-1 could modulate the cell migrative and invasive ability of OS cells. A Transwell chamber assay was performed to detect the invasive activity of OS cells after Notch-1 shRNA transfection. We observed that the suppression of Notch-1 reduced the invasive ability of U2OS and MG63 cells (Figure 1C). Accordingly, a wound healing assay was employed to examine the migrative ability of OS cells after Notch-1 downregulation. Our data demonstrated that in OS cells, Notch-1 shRNA infection led to a reduction in wound closure compared with that of the control group (Figure 2A, 2B). In summary, Notch-1 inhibition reduced the migration and invasion of OS cells.

### Inhibition of Notch-1 reduces Cdc20 expression

To explore the mechanism of Notch-1-mediated oncogenesis, the expression of Cdc20 in OS cells after Notch-1 shRNA transfection was evaluated using real-time RT-PCR and western blotting. We found that the inhibition of Notch-1 reduced Cdc20 expression levels at mRNA and protein in U2OS and MG63 cells after Notch-1 shRNA transfection (Figure 2C, 2D). The expression of two downstream targets of Cdc20, Bim and p21, was alleviated in OS cells after Notch-1 shRNA transfection (Figure 2C, 2D). These results indicate that Notch-1 exhibits its function partly through modulating Cdc20 and its downstream genes in OS cells.

### Notch-1 upregulation enhances viability and represses apoptosis

**Figure 3:**
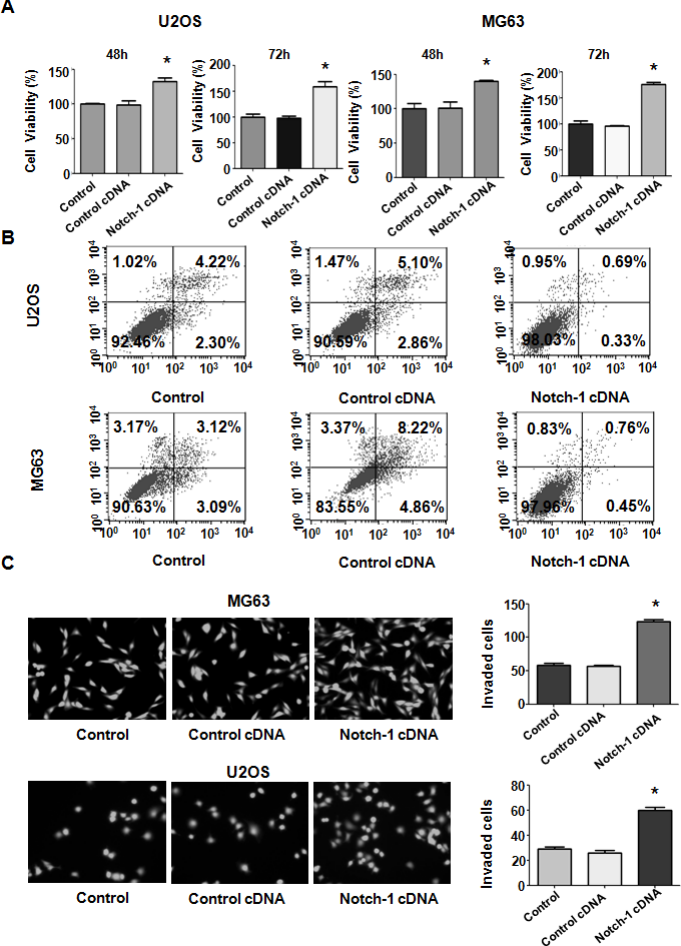
**Notch-1 upregulation elevated viability and suppressed apoptosis.** (**A**) Viability was elevated after Notch-1 upregulation, as determined by MTT assay. *P < 0.05 vs control cDNA. (**B**) Apoptosis was inhibited after Notch-1 upregulation. (**C**) Invasion was enhanced after Notch-1 upregulation, as determined by Transwell assay.

To further investigate the biological function of Notch-1 in OS cells, a Notch-1 cDNA vector was transfected into U2OS and MG63 cells. The MTT assay was employed to examine the viability of OS cells after Notch-1 cDNA vector transfection. Notch-1 upregulation led to the promotion of viability in U2OS and MG63 cells (Figure 3A). These MTT data suggest that Notch-1 overexpression facilitates the viability of OS cells. In addition, the apoptosis rate of OS cells after Notch-1 cDNA vector transfection was determined by a PI-FITC-annexin assay. Notch-1 overexpression repressed apoptosis in both U2OS and MG63 cells (Figure 3B). Apoptosis was alleviated from 8% to 1% in U2OS cells in Notch-1 cDNA group (Figure 3B). A similar trend in apoptosis reduction was observed in MG63 cells (Figure 3B). Taken together, these results suggest that Notch-1 upregulation enhances cell viability and represses apoptosis.

### Upregulation of Notch-1 facilitates cell motility

**Figure 4:**
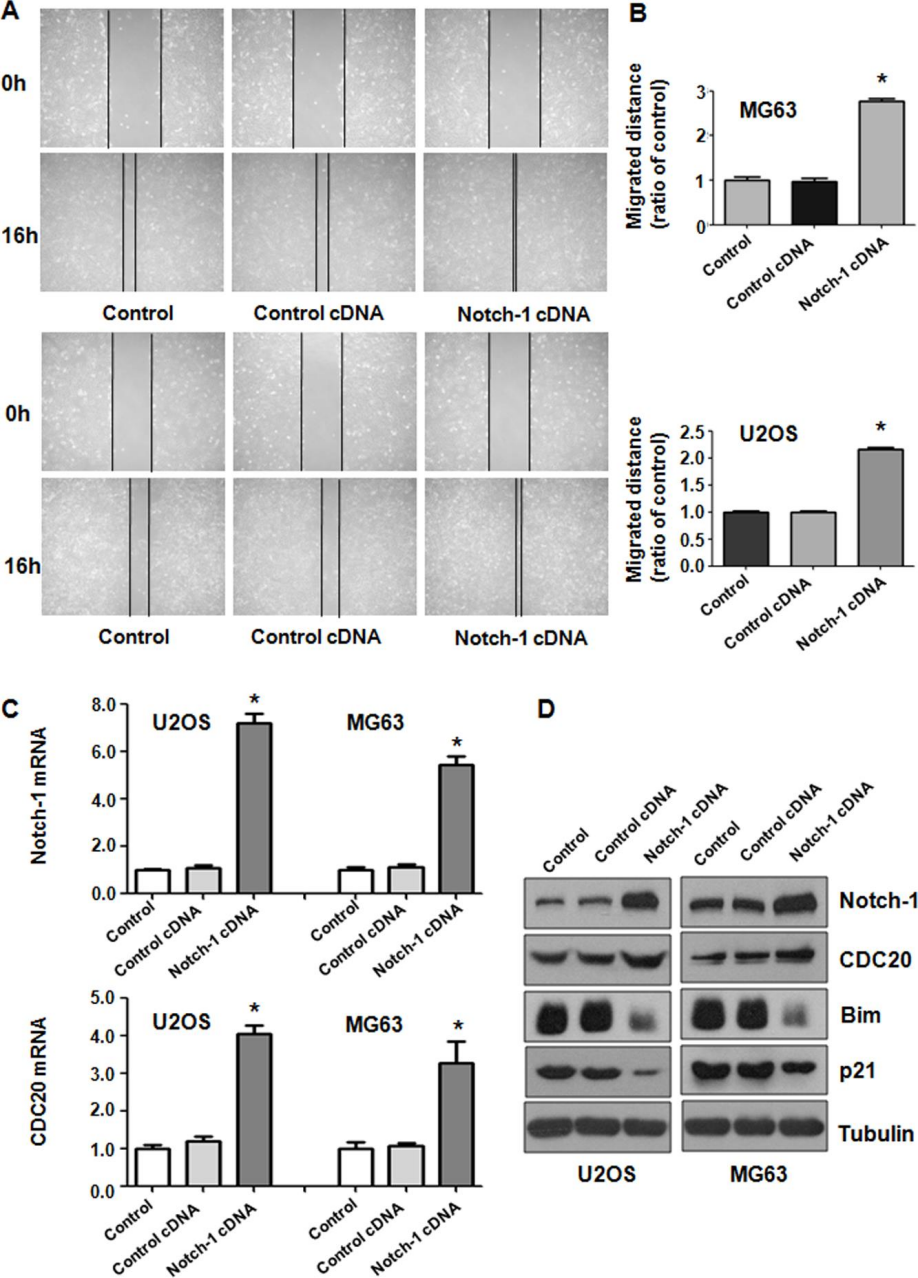
**Notch-1 overexpression elevated Cdc20 expression.** (**A**) The migratory ability was promoted by Notch-1 overexpression. (**B**) Quantification of the migration analysis results. *P < 0.05 vs control cDNA. (**C**) Real-time RT-PCR showing Notch-1, Cdc20, p21 and Bim expression in cells with Notch-1 upregulation. (**D**) Western blot results showing Notch-1, Cdc20, p21 and Bim expression in cells with Notch-1 upregulation.

To further test the function of Notch-1 in governing the invasive ability of OS cells, a Transwell assay was carried out to analyze the invasiveness of U2OS and MG63 cells after Notch-1 overexpression. We found that the upregulation of Notch-1 increased the number of invading U2OS and MG63 cells (Figure 3C). The wound healing assay was applied for examining the migrative activity of OS cells overexpressing Notch-1. The data showed that Notch-1 overexpression facilitated the wound closure rate of MG63 and U2OS cells (Figure 4A, 4B). In summary, Notch-1 upregulation facilitated the motility of OS cells.

### Notch-1 overexpression increases Cdc20 expression

Western blotting analysis was utilized to examine Cdc20 expression in OS cells after Notch-1 upregulation. Real-time PCR and Western blot data showed that Notch-1 overexpression elevated Cdc20 expression levels in U2OS and MG63 cells (Figure 4C, 4D). In line with Bim and p21 being downstream factors of Cdc20, we saw that the overexpression of Notch-1 diminished Bim and p21 protein levels in OS cells (Figure 4C, 4D). These results revealed that Notch-1 overexpression increased Cdc20 expression in OS cells and subsequently attenuated the expression of Bim and p21.

### Overexpression of Cdc20 rescues the impaired viability caused by Notch-1 downregulation

**Figure 5:**
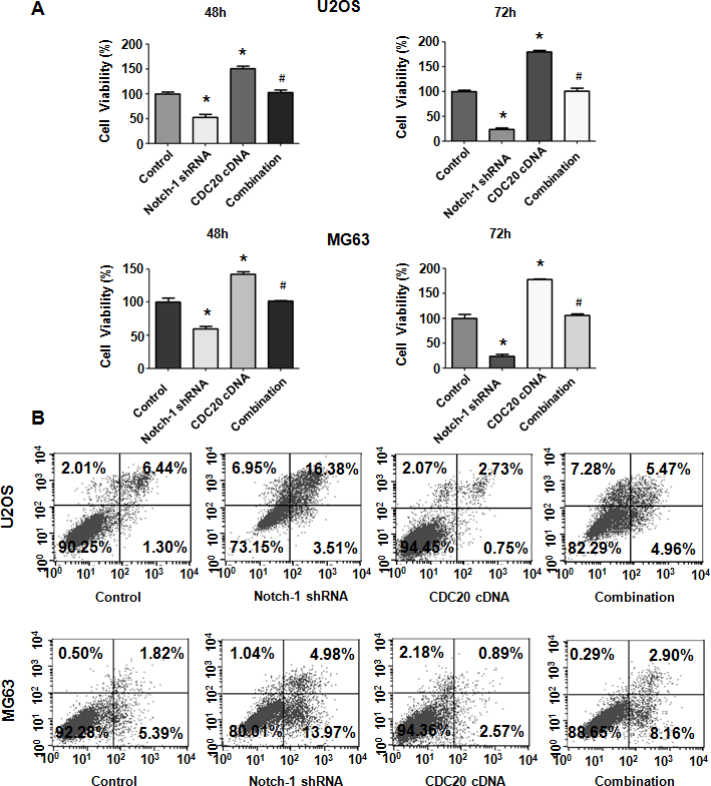
**Cdc20 overexpression abrogated Notch-1 shRNA-mediated antineoplastic effects.** (**A**) Viability was examined in cells cotransfected with Notch-1 shRNA and Cdc20 cDNA. *P < 0.05 vs control shRNA, ^#^ P < 0.05 vs Notch-1 shRNA or Cdc20 cDNA. (**B**) Apoptosis was examined in osteosarcoma cells after Notch-1 shRNA and Cdc20 cDNA cotransfection.

To define whether Notch-1 performs its oncogenic function via the upregulation of Cdc20 in OS cells, rescue experiments were done. After OS cells were cotransfected with both Notch-1 shRNA and the Cdc20 cDNA vector, an MTT assay was employed to examine the viability of U2OS and MG63 cells. Our results revealed that Cdc20 upregulation facilitated the viability of OS cells (Figure 5A). Moreover, the overexpression of Cdc20 abrogated the reducing in viability induced by Notch-1 shRNA transfection (Figure 5A). Therefore, Notch-1 governs the viability of OS cells in part via the upregulation of Cdc20.

### Cdc20 overexpression abrogates apoptosis induced by Notch-1 downregulation

Next, OS cell apoptosis was evaluated after Notch-1 shRNA and Cdc20 cDNA cotransfection. Cdc20 upregulation repressed apoptosis of MG63 and U2OS cells (Figure 5B). Notably, Cdc20 upregulation abrogated Notch-1 downregulation-induced apoptotic death (Figure 5B). Our results demonstrated that Notch-1 inhibition promoted apoptosis via the reduction in Cdc20 in OS cells.

### Overexpression of Cdc20 blocks Notch-1 downregulation-mediated migration.

**Figure 6:**
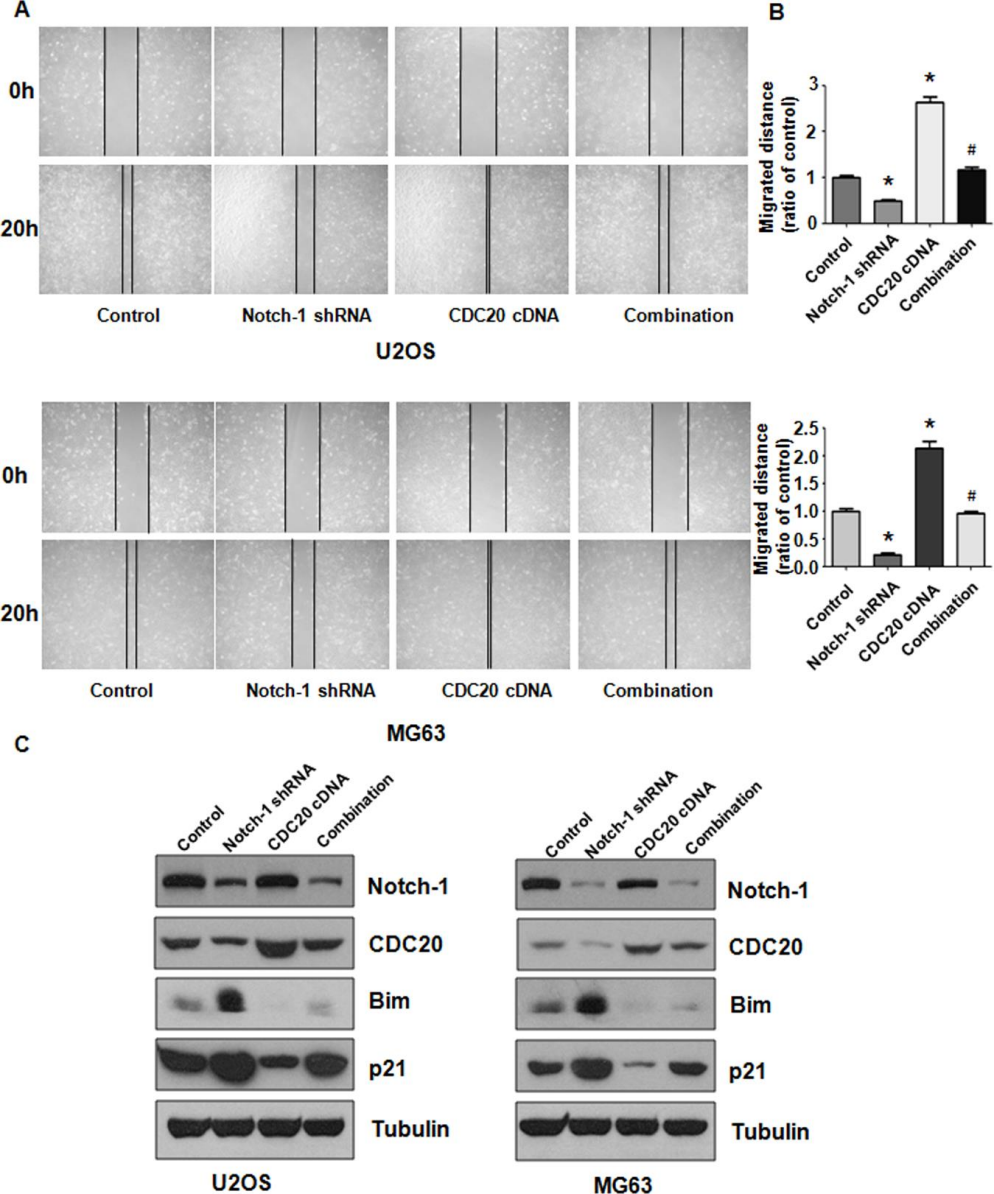
**Cdc20 overexpression abolished shRNA-mediated Notch-1 suppression.** (**A**) The wound healing assay showed that Cdc20 upregulation abolished Notch-1 downregulation-mediated suppression of viability. (**B**) Quantification of the migration analysis results. *P < 0.05 vs control shRNA, ^#^ P < 0.05 vs Notch-1 shRNA or Cdc20 cDNA. (**C**) Western blot results showing Notch-1, Cdc20, Bim and p21 expression.

The migratory ability of OS cells was examined after Notch-1 shRNA and Cdc20 cDNA vector cotransfection. As expected, Cdc20 upregulation elevated the migration of OS cells (Figure 6A, 6B). Furthermore, Cdc20 upregulation blocked Notch-1 downregulation-mediated inhibition of wound closure (Figure 6A, 6B). Mechanistically, increased Cdc20 expression abrogated the Notch-1 shRNA-mediated inhibition of Cdc20 expression in OS cells (Figure 6C). Strikingly, the overexpression of Cdc20 abrogated the upregulation of Bim and p21 triggered by Notch-1 shRNA transfection in OS cells (Figure 6C). In addition, Cdc20 overexpression did not change Notch-1 expression levels (Figure 6C), indicating that Notch-1 is an upstream factor of Cdc20.

## DISCUSSION

A line of evidence has implicated a crucial role of Notch-1 in OS development and progression. OS cells with increased expression of Notch-1, Notch-2, DLL1 and Hes-1 exhibited a high ability to metastasize [22]. Moreover, GSI treatments led to the suppression of the invasiveness of OS cells. Strikingly, DAPT inhibited ERK phosphorylation and alleviated proliferation and metastasis in OS cells [23]. Furthermore, Hes-1 expression was inversely associated with survival in OS patients [22]. Interestingly, Hes-1 and Deltex1 also reciprocally inhibited Notch signaling to attenuate OS invasiveness [24]. These reports indicate that the Notch signaling pathway is involved in OS metastasis [22]. In the present study, we identified that Notch-1 plays an oncogenic role in OS, including promoting cell viability, migrative and invasive ability, and suppressing apoptosis.

Several studies have revealed the molecular mechanism of the Notch pathway in OS tumorigenesis. For example, the Notch pathway activates OS progression via the upregulation of ephrinB1 [25]. Our study dissected that Notch-1 activates Cdc20 expression and inhibits Bim and p21 expression in OS cells. Moreover, Notch-1 gains its oncogenic function via the upregulation of Cdc20 in OS.

Therefore, targeting Notch-1 might be an effective treatment for OS patients with increased levels of Notch-1. Multiple compounds have been identified that suppress the activation of Notch-1 in OS. For instance, curcumin, a phenolic agent derived from Curcuma longa, repressed

the proliferation and invasion of OS cells by blocking Notch-1 signaling [26]. Diallyl trisulfide suppressed proliferation, invasion and angiogenesis via the inactivation of Notch-1 and activation of suppressor miRNAs in OS cells [27]. Oleanolic acid stimulated OS cell apoptosis via the suppression of the Notch signaling pathway [28]. Resveratrol inhibited OS progression via the upregulation of miR-139 and subsequent downregulation of Notch-1 in OS cells [29]. It is necessary to explore whether these natural agents could inhibit the expression of both Notch-1 and Cdc20 in OS cells.

Studies have also demonstrated that noncoding RNAs regulate Notch-1 in OS cells. One group reported that miR-34a repressed cell proliferation and metastasis in OS cells via targeting the Notch signaling pathway [30]. Moreover, miR-34a exerts a combinational effect with celecoxib in the regulation of the proliferation and invasiveness of OS cells by targeting Notch-1 [31]. Another study revealed that miR-199b-5p participated in osteosarcoma progression via the regulation of Notch pathways [32]. In addition, miR-135b elevated OS distant metastasis and recurrence via the activation of the Notch and WNT/β-catenin pathways [33]. The overexpression of miR-340 enhanced OS cell apoptosis, and inhibited proliferation and motility via the repression of the Notch signaling pathway [34]. Another study revealed that miR-1296-5p repressed the growth, migration and invasion of OS cells by targeting Notch-2 [35]. Moreover, miR-92a blocked OS progression and induced cisplatin sensitivity via the inhibition of Notch-1 [36].

Recently, lncRNA MEG3 was shown to repress proliferation, stimulate apoptosis in OS cells and block metastasis by reducing Notch-1, Jagged-1, Hes-1 and NICD1 [37, 38]. Similarly, lncRNA CEBPA-AS1 upregulation suppressed proliferation and motility and triggered apoptosis by diminishing the Notch pathway [39]. LncRNA CRNDE increased the proliferation and migrative and invasive ability of OS cells via the modulation of Notch-1 and EMT [40]. LncRNA SNHG7 facilitated tumor growth and EMT by targeting the miR-34a and Notch pathways in OS [41]. LncRNA NBR2 suppressed cell proliferation, invasion and migration and EMT via suppressing Notch-1 in OS cells [42]. Therefore, how Notch-1 is regulated by noncoding RNAs in OS cells needs to be further explored in the near future. In summary, Notch-1 promoted the viability and motility of OS cells by upregulating Cdc20 in OS cells.

## MATERIALS AND METHODS

### Transfection

MG63 and U2OS cells were grown in complete DMEM. OS cells were cultured overnight and transfected with Notch-1 cDNA, empty vector, control shRNA, Notch-1 shRNA or Cdc20 cDNA or a combination by Lipofectamine 2000 according to the instructions as previously described [20]. The CDC20 shRNA plasmid DNA (SHCLND-NM-001255) was purchased from Sigma Aldrich Company, and Notch1 shRNA plasmid (SC-36095-SH) was obtained from Santa Cruz Biotechnology Company. The transfected cells were further analyzed for biological functions.

### Cell viability

The transfected OS cells were incubated in 96-well plates for 48 hours and 72 hours. The MTT assay was utilized to measure the viability of OS cells as previously described [21].

### Cell apoptosis

The transfected OS cells were incubated in six-well plates for 72 hours. Cells were collected and resuspended in binding buffer with propidium iodide and FITC-conjugated anti-Annexin V antibodies. The apoptosis rate was determined as described before [21].

### Cell invasion assay

The transfected OS cells were incubated in the top chamber of Transwell insert with serum-free medium. The bottom chamber was filled with complete medium. After 20 hours of incubation, calcein AM was employed to stain the invading cells for 1 hour. The invading cells were photographed by a microscope.

### Wound healing assays

The transfected OS cells were incubated in 6-well plates until the cells covered the well. A yellow pipette tip was used to create a wound in the OS cells. After 16 hours and 20 hours, the wound area was photographed with a microscope.

### Real-time quantitative reverse transcription PCR (RT-qPCR)

Total RNA from transfected cells was extracted using TRIzol reagent, and RT-PCR was performed as described previously [43].

### Western blotting analysis

The cells were lysed by protein lysis buffer after 72 hours post-transfection with cDNA or shRNA. The protein levels were examined by western blotting. The primary antibodies were incubated with the membranes at 4° C overnight. The expression level of target proteins was evaluated as described previously [20].

### Statistical analysis

All data were analyzed by GraphPad Prism 5.0. ANOVA was utilized to analyze statistical significance. Each experiment was performed in triplicate. A value of P < 0.05 was considered statistically significant.
